# Prediction and diagnosis of depression using machine learning with electronic health records data: a systematic review

**DOI:** 10.1186/s12911-023-02341-x

**Published:** 2023-11-27

**Authors:** David Nickson, Caroline Meyer, Lukasz Walasek, Carla Toro

**Affiliations:** 1 WMG, University of Warwick, Coventry, UK; 2 Warwick Medical School, University of Warwick, Coventry, UK; 3 Department of Psychology, University of Warwick, Coventry, UK

**Keywords:** Artificial Intelligence, Depression, Diagnosis, Electronic Health Records, Machine Learning, Prediction

## Abstract

**Background:**

Depression is one of the most significant health conditions in personal, social, and economic impact. The aim of this review is to summarize existing literature in which machine learning methods have been used in combination with Electronic Health Records for prediction of depression.

**Methods:**

Systematic literature searches were conducted within arXiv, PubMed, PsycINFO, Science Direct, SCOPUS and Web of Science electronic databases. Searches were restricted to information published after 2010 (from 1st January 2011 onwards) and were updated prior to the final synthesis of data (27th January 2022).

**Results:**

Following the PRISMA process, the initial 744 studies were reduced to 19 eligible for detailed evaluation. Data extraction identified machine learning methods used, types of predictors used, the definition of depression, classification performance achieved, sample size, and benchmarks used. Area Under the Curve (AUC) values more than 0.9 were claimed, though the average was around 0.8. Regression methods proved as effective as more developed machine learning techniques.

**Limitations:**

The categorization, definition, and identification of the numbers of predictors used within models was sometimes difficult to establish, Studies were largely Western Educated Industrialised, Rich, Democratic (WEIRD) in demography.

**Conclusion:**

This review supports the potential use of machine learning techniques with Electronic Health Records for the prediction of depression. All the selected studies used clinically based, though sometimes broad, definitions of depression as their classification criteria. The reported performance of the studies was comparable to or even better than that found in primary care. There are concerns with generalizability and interpretability.

**Supplementary Information:**

The online version contains supplementary material available at 10.1186/s12911-023-02341-x.

## Background

Depression is the most common mental health condition globally, with one-year global prevalence rates ranging from 7 to 21% [1]. Quality of life can be seriously impaired by this disorder, with depression ranking as the second highest cause of Disability-Adjusted Life Years (DALYs) and Years Lived with Disability (YLDs) [2, 3]. Depression is a major contributory factor in suicide affecting hundreds of thousands of cases per year [4, 5]. In addition to the significant personal and social impact of depression, there is a significant economic cost. For example, in 2007 alone, total annual costs of depression in England were £7.5 billion, of which health service costs comprised £1.7 billion and lost earnings £5.8 billion [6, 7]. More recently, in 2019, it was estimated that mental health problems cost the UK £ 118 billion per year, of which 72% were due to lost productivity and other indirect costs. At 22% prevalence depression was identified as the third highest contributor to these costs [8, 9].

Depression, like most mental health disorders, can be difficult to diagnose, especially for non-specialist clinicians [10, 11]. Assessment by primary or secondary care clinicians typically relies on the World Health Organisation’s International Catalogue of Diseases version 10 or 11, ICD-10/11 [12], the Diagnostic and Statistical Manual of Mental Disorders DSM [13], or by using an interview script such as the Composite International Diagnostic Interview (CIDI) [14, 15]. Diagnosis can also be aided by garnering self-reported symptoms in response to standardised questionnaires such as the Hospital Anxiety and Depression Scale (HADS) [16], Beck Depression Inventory (BDI) [17, 18] and Patient Health Questionnaire-9 (PHQ-9) [19, 20]. The PHQ-9 is considered a gold standard [21] for screening rather than standalone clinical diagnosis [22] and has been validated internationally [20]. As such it sets a sound benchmark for sensitivity (e.g., 0.92) and specificity (e.g., 0.78) that is a good comparator for assessing alternative methods [23].

Considering mental health care pathways, benefits to patients could be provided by early diagnosis, opening the possibility to early interventions. For example, Bohlmeijer et al. [24] observed reduced symptoms of depression for patients who engaged in acceptance and commitment therapy (ACT) as an early intervention compared to those on a wait list, both initially and at a three month follow up. Furthermore, a meta-analysis by Davey and McGorry [25] showed a reduction in the incidence of depression by about 20% in the 3 to 24 months following an early intervention. At the same time, late diagnoses of depression can result in longer term suffering for the patient in terms of symptoms experienced and disorder trajectory together with increased resource consumption [10, 26].

Recently, attempts to support early medical diagnoses have benefited from a) growing availability of electronic healthcare records (EHRs) that contain patients’ longitudinal medical histories and b) new advances in predictive modelling and machine learning (ML) approaches. The use of EHRs in primary care in the developed world is well established. For example, in the USA, UK, Netherlands, Australia and New Zealand, take up in primary care has exceeded 90% [27, 28]. The wide availability of proprietary EHR systems such as SNOMED (Systematized Nomenclature For Medicine) in the UK [29] are enabling rapid and global implementation and their use for disorder surveillance [30]. For example, ML techniques with EHR data have led to predictive models for cardiovascular conditions [31, 32] and diabetes [33]. These studies have led to cardiovascular risk prediction becoming established in routine clinical care and the UK QRISK versions 2 and 3 show significant improvements in discrimination performance over the Framingham Risk Score and atherosclerotic cardiovascular disease (ASCVD) score methods [34] that preceded them. Many of the recent advances were facilitated by the growing popularity of ML in medical data science. As a subfield of artificial intelligence (AI), ML allows computers to be trained on data to identify patterns and make predictions. This approach is well suited for developing algorithms to predict the likelihood of a patient having a disorder by analysing large volumes of medical data. Once trained, these algorithms can then be tested on new data to assess their performance outside of the training environment. There are a variety of ML techniques, but the two most common include supervised and unsupervised methods. In supervised learning data are labelled with desired outcome. In unsupervised learning the data are not labelled, and the algorithms look for patterns within the data without external guidance. Further information on these methods in relation to mental health and EHRs is provide in Cho et al. [35] and Wu et al. [36] but here we note that many existing applications combine some unsupervised and supervised methods to train algorithms on datasets with large numbers of predictors. A scoping review by Shatte et al. [37] on the general use of ML in mental health identified the use of ML with EHRs for identifying depression as a research area. Similarly, Cho et al. [35] included depression amongst the conditions they identified in their “Review of Machine Learning Algorithms for Diagnosing Mental Illness”. In the examples they cite, which are also covered in the results of this systematic review, ML algorithms were trained on EHRs data that included a variety of symptoms and conditions. These algorithms were then assessed on their ability to distinguish between those who did/did not have clinical depression. If EHR/ML methods are to be considered, a suitable benchmark comparator is needed. Studies assessing diagnosis of depression in primary care suggest that approximately half of all cases are missed at first consultation but that this improves to around two thirds being diagnosed at follow up [38–40]. This would be a useful minimum comparator for any diagnostic system based on a combination of ML and EHRs data. There exists the potential to develop predictive models of depression using EHR/ML applications and it is necessary to critically evaluate models developed in recent years. This is particularly important in the context of rapidly developing ML techniques, and the growing accessibility and richness of EHRs health data. Our starting point for this systematic review was, “Is there a case for using EHRs with machine learning to predict/diagnose depression?” From this we derived the objectives to identify and evaluate studies that have used such techniques. As part of the evaluation, we specifically focus on identifying key features of the data and ML methods used. Accordingly, our primary focus is to provide a comprehensive overview of the types of ML models and techniques used by researchers, as well as types of data on which these models were trained, how the models were validated and, where done, how they were then tested. By summarizing the data used, identifying and summarising predictors used, describing diagnostic benchmarks, and outlining what types of validation and testing approaches were used, our review offers an important source of information for those who wish to build on existing efforts to improve predictive accuracy of such models.

## Methods

### Search strategy and search terms

Systematic literature searches were conducted within arXiv, PubMed, PsycINFO, Science Direct, SCOPUS and Web of Science electronic databases. Searches were restricted to information published after 2010 (from 1^st^ January 2011 onwards) and were updated prior to the final synthesis of data on 27^th^ January 2022. Initial searches were made based on titles/key words (where latter available) and papers were selected based on the inclusion criteria summarised in Table 1. These were searched as (#1) AND (#2) AND (#3) AND (#4). These papers were evaluated by reading the Abstract, and then by evaluating main body of each manuscript. Next, a backward citation search for all the selected papers was completed as both a) a quality check to see if other selected papers were included and b) to identify any missing papers. The last search step was a forward search pass where papers that cited the selected papers were identified; again, identifying any missed papers. The same time period and inclusion/exclusion criteria were applied to these additional searches. The initial searches together with primary assessment for inclusion were conducted by DN. 10% of the searches were sampled by LW. The inclusion/exclusion results for the selected papers were audited by LW, and joint discussions were held to resolve any issues. In the event of this not being possible CT would have been involved as final arbiter.

**Table 1:** Search terms for study identification

*Component*	*Area*	*Search terms*
#1	Artificial Intelligence/Machine Learning	(artificial intelligence) OR (machine learning) OR (data mining) OR (supervised learning) OR (unsupervised learning) OR (predictive analytics) OR (reinforcement learning) OR deep learning)
#2	Screening/Diagnosis	(screening, including: screen*; identif* detect*) OR (diagnosis including diagnos*) OR (Classification) OR (prediction including: predict*)
#3	Depression	Depression OR Depressive
#4	Electronic Health Records	(Electronic Health Records, including EHR) OR (Electronic Medical Records, including EMR) OR (Clinical records) OR Clinical notes)

Note 1, The symbol "*" in search terms indicates match the core text followed by any valid suffix , e.g., "ing"

This systematic review was prospectively registered with Prospero international database of systematic reviews (# CRD42021269270) [41].

### Inclusion/exclusion criteria

Table 2 shows the inclusion and exclusion criteria that were adopted to define the publications that came within the scope of the review.

**Table 2:** Inclusion/exclusion criteria

*Inclusion*	*Exclusion*
Screening/Prediction/Diagnosis of depression in the undiagnosed with/without comorbidities	Involved interventions/trials or delivery/monitoring of interventions
Artificial Intelligence/Machine Learning techniques	Used additional unproven, experimental, bespoke or laboratory technology
Used EHRs/Clinical notes derived data as primary source	Used additional high cost/specialist technology such as fMRI (functional Magnetic Resonance Imaging**)** scanners, ECG (electrocardiogram), EEG (electroencephalogram)**,** PET (Positron Emission Tomography) scans, radiography etc
Ethically approved	Involved invasive procedures such as blood tests, CSF (Cerebrospinal Fluid) assays
Took place after 01/January/2011	Required additional activity to obtain predictor data e.g., clinical interviews
Available in English	Review/Summary paper
Published in a peer reviewed journal/recognised publisher/conference paper	

### Data extraction

Data extraction was informed by requirements detailed in: ‘Transparent reporting of a multivariable prediction model for individual prognosis or diagnosis (TRIPOD) [42]; ‘Critical Appraisal and Data Extraction for Systematic Reviews of Prediction Modelling Studies: The CHARMS Checklist’ [43], and ‘Protocol for a systematic review on the methodological and reporting quality of prediction model studies using machine learning techniques’ [44]. Table 3 details the data extraction categories. Primary data extraction was conducted by DN this was then validated by LW.

**Table 3:** Data extraction summary

*Category*	*Description/example*
Title	Title of journal/conference entry
Journal/ Conference	Publisher
Outcome Benchmark for depression	How outcome was measured (e.g., PHQ-9 (Patient Health Questionnaire 9), ICD (International Classification of Diseases) code, HADS (Hospital Anxiety and Depression Scale)
Demographic	Characteristics of the participant pool including age, gender, ethnicity etc. where specified
Data Source type	EHRs (Electronic Health Records), EMRs (Electronic Medical Records), Clinical Notes, Clinical Records
Data Specifications	Nature and source of data (e.g., types of codes used, organisation that provided the data)
Predictors	Types of predictors used by models and identification of any groupings or subsets they might fall into. The term “predictors” is considered interchangeable with “features” and “exposure variables” or other related terms
Study Design	Case/Control, Case Series, Cohort etc
Sample Size Training or Total	Number included in training/total dataset
Sample Size Testing/Validation	Number included in test/validation dataset
Missing Data	Explanation of how instances of missing data were addressed
Model Development Pre-Process	Information relating to the methods used for pre-processing, preparing, cleaning, extracting data (e.g., natural language and text processing methods)
Model Development Analysis (Fitting)	Information relating to the statistical methods used, ML (statistical techniques and/or broader AI e.g., neural networks). If relevant additional data pre-processing/preparation. Assessment of overfitting
Performance Metric	How model measured/reported (e.g., odds ratio, AUC ROC (Area Under Curve Receiver Operating Characteristic, Sensitivity, Specificity, Accuracy)
Baseline/Comparator	Criteria used to evaluate/compare model. How model assessed against outcome
Validation	Information relating to the use of validation methods
Testing	Independent testing and separate hold out set
Results	The results reported (may be in summary form)
Data Availability and sharing	Information relating to data availability, any repository/contact details and conditions that might apply
Code Availability and sharing	Information relating to code availability, any repository/contact details and conditions that might apply
Abstract	Text of study abstract
Full Reference (and Citation)	Supporting unambiguous identification of paper and providing source for citations in tables/figures/text

### Quality of studies

The Oxford Centre for Evidence-Based Medicine (OCEBM) system [45] was used to assess quality, previously used for a systematic review about artificial intelligence and suicide prevention by Bernert et al. [46] as many of the models were developed and evaluated in a clinical setting and so merit a level of formal assessment. This ranked the evidence on a scale of 1 to 5, lowest to highest. The results were added to the data extraction table. OCEBM is designed to provide a hierarchy of levels of evidence for researchers and clinicians whose time is limited, it is well established and widely used. For further information, see Howick et al. as reported in [47].

## Results

**Fig. 1:**
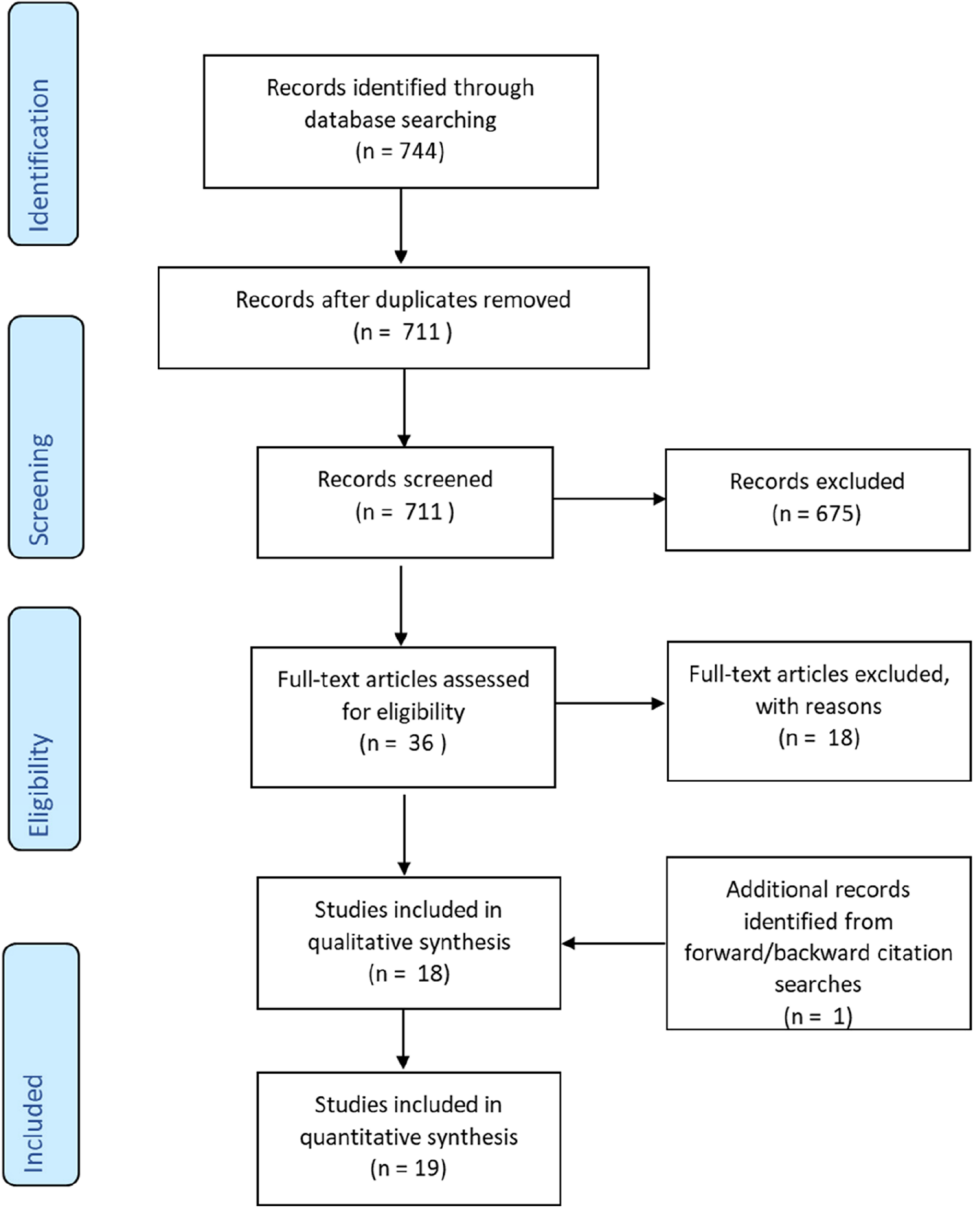
PRISMA flow diagram with results for systematic review study selection [48]. Note: reasons, for example relating to disorder focus, scope, data sources, specially selected cohorts, disorder trajectory not diagnosis, for excluding full text articles are included in supplementary data, Table S 1

The search protocol together with numbers of studies identified, selected, assessed, included/excluded is presented in Fig. 1, compatible with PRISMA standard [48].

### Searches

A total of 744 research papers were identified in the first stage of the literature search (711 after duplicates were removed). Screening content of abstracts and, subsequently, main body of each article, reduced the sample to 18 eligible articles. The backwards citation search of the selected papers identified 22 papers (including duplicates) that were rejected, 10 that were in the original selection and two (duplicates) that were added to the selection, resulting in one additional paper (giving 19 in total). The forward citation search did not produce additional papers at the time of the review.

Review articles are not included in the final total but were used for supporting research and were recorded.

### Selected studies overview

This review summarised studies that use ML methods to train validate, and test ML models for predicting depression based on individual-level EHR data from primary care (11 studies) and from a combination of primary and secondary care (8 studies). Table 4 summarizes key features of each study. We now turn to a detailed overview of each of the components described in Table 4.

**Table 4:** Methods, performance, demographics, evaluation summary for the 19 selected papers [49–67]

**Citation**	**Outcome Benchmark for Depression**	**Demographic**	**Data Source**	**Data Specifications**	**Data Sharing**	**Study Design**	**Predictors** (Note 1)					
			**(Primary, Secondary, Study collected)**			**(Case Control, Case-series, Cross-Sectional, Historical Control,..)**	**Comorbidities**	**Demographic**	**Family History**	**Obstetric specific**	**Psychiatric**	**Smoking**
Abar et al. [49]	International Classification of Diseases version 9 (ICD-9) Codes	University of Kentucky (UKY) medical center Electronic Health Records (EHRs). All patient visits during the ten year period 2004–2013. Mixed ethnicity USA assumed, details unspecified	Primary, Secondary	3 million patient visits to the University of Kentucky (UKY) medical center and its affiliated clinics. The dataset has 11,877 unique International Classification of Diseases, Clinical Modification, Version 9 (ICD-9-CM) codes and 1032 unique medication codes by Cerner Multum™LexiconPluscodes	Not offered. Source identified	Cohort	✓		✓	✓	✓	✓
Geraci et al. [50]	Clinical Psychiatrists(2), Diagnostic and Statistical Manual of Mental Disorders-IV (DSM)-IV depression	Aged 12 to 18, 60% female, 40% male. 861 individuals from Centre for Addiction and Mental Health, Toronto, ON M6J1H4. Assumed mixed ethnicity (not specified) representative of Canada	Primary	EHRs format not specified	Not offered. Source identified	Cohort					✓	
Hochman et al. [51]	ICD 9, ICD 10 or antidepressant (WHO Anatomical Therapeutic Chemical [ATC] code N06A) (thus excluding off‐label use)	A nationwide longitudinal cohort that included 214,359 births between January 2008 and December 2015. Israel. Average age 29.4 (SD, 5.4) for training set, 29.8 (SD 5.5) validation set. Mixed Arab (circa 34%) Jewish (circa 66%) ethnicity	Primary, Secondary	Clalit Health Services (CHS) EHR data warehouse. ICD‐9 or ICD‐10 codes recorded in the EHRs	The raw data used for this study will be stored at the Clalit servers and within its firewall, and will be made available upon request under the limitations and requisites of the Clalit regulations and Israeli Privacy Laws	Cohort	✓ (5)	✓ (5)		✓ (21)	✓ (4)	✓ (1)
Huang et al. [52]	Patient Health Questionnaire 9 (PHQ-9), ICD-9	Age 18 + , EHR data from the Palo Alto Medical Foundation (PAMF) 55.2% female, mixed ethnicity. Group Health Research Institute (GHRI) 70.3% female, mixed ethnicity	Primary	Epic EHR system. International Classification of Diseases, Ninth Revision (ICD-9) diagnosis codes, RxNorm prescription codes, and Current Procedural Terminology (CPT) procedure codes; and unstructured data such as progress notes, pathology reports, radiology reports, and transcription reports. All structured and unstructured data are time-stamped	Not offered. Source identified	Cohort		✓ (4)	✓ (1)		✓ (7)	
Jin et al. [53]	PHQ-9, PHQ-8 item 9	Predominantly Latino diabetes patients within a USA public safety net care system: 62% age 45 and older; 68% female	Primary, Secondary	Diabetes-Depression Care-management Adoption Trial (DCAT), a comparative effectiveness study from 2010 to 2013 with three arms: Usual Care (UC) Los Angeles County Department of Health Services (LACDHS), Supported Care (SC), and Technology Care (TC)	Not offered. Source (DCAT) identified	Cohort	✓	✓				
Kasthurirathne et al. [54]	Physician assessed. ICD-9 and ICD-10 codes	USA sample. Mixed ethnicity. 84,317 adult patients (≥ 18 years of age) with at least 1 primary care visit between the years 2011 and 2016 at Eskenazi Health, Indianapolis, Indiana. Average Age 43.88 (SD 15.60), male 35.09%, White (non-Hispanic) 25.21%, African American (non-Hispanic) 37.23%, Hispanic or Latino 19.47%	Primary	Indiana Network for Patient Care (INPC), structured International Classification of Diseases, ninth revision (ICD-9) and ICD-10 codes. The dataset included a wide array of patient data, including patient demographic, diagnostic, behavioral, and visit data reported in both structured and unstructured form	Not offered. Source (INCP) identified	Cohort	✓	✓			✓	
Koning et al. [55]	Mental Health Problem by WHO International Classification of Primary Care (ICPC) and ATC Code including depression	Patients aged 1–19 years on 31 December 2016 without prior mental health problems. 76 general practice centres in the Leiden area of the Netherlands. Representative of local population	Primary	ELAN primary care network (Extramural Leiden Academic Network) of the Leiden University Medical Centre (LUMC), the Netherlands. Patient data included demographics, consultation dates, symptoms and diagnoses coded according to the WHO International Classification of Primary Care (ICPC), prescribed medication coded according to the Anatomical Therapeutic Chemical (ATC) classification, laboratory test results, and descriptive or coded information of referrals and correspondence with other healthcare professionals	Not offered. Source identified	Cohort	✓	✓	✓			
Meng et al. [56]	Depression related ICD-9 codes, inclusion of an antidepressant drug in a patient’s medication list, or appearance of an antidepressant drug in clinical notes	EHRs source not specified. Patients selected based on three primary diagnoses: myocardial infarction (MI), breast cancer, and liver cirrhosis. Generally, MI represents the least complexity, cirrhosis the most. 68.78 SD ± 15.46. Min 18, max, 98. Male 27.46%, female 72.54%	Primary, Secondary	International Classification of Disease, ninth revision (ICD-9) format, procedure codes in Current Procedural Terminology (CPT) format, medication lists, demographic information, and clinical notes	Not offered. Source not identified	Cohorts(3)		✓			✓	
Meng et al. [57]	Depression related ICD-9 code, inclusion of an antidepressant drug in a patient's medication list, appearance of an antidepressant drug in clinical notes	EHRs source not specified. Patients selected based on three primary diagnoses: myocardial infarction (MI), breast cancer, and liver cirrhosis. Generally, MI represents the least complexity, cirrhosis the most. 68.78 SD ± 15.46. Min 18, max, 98. Male 27.46%, female 72.54%	Primary, Secondary	International Classification of Disease, ninth revision (ICD-9) format, procedure codes in Current Procedural Terminology (CPT) format, medication lists, demographic information, and clinical notes	Not offered. Source not identified	Cohorts (3)		✓			✓	
Nemesure et al. [58]	DSM-IV	Students. University of Nice Sophia-Antipolis. Ages, under 18 to over 20. Gender and French nationality status	Primary, Study data	CALCIUM database (Consultations Assistés par Logiciel pour les Centres Inter-Universitaire de Médecine) and included information about the students’ lifestyle (living conditions, dietary behavior, physical activity, use of recreational drugs)	Yes, all data, de-identified, was publicly available on Dryad a nonprofit membership organization that is committed to making data available for research and educational reuse now and into the future. https://datadryad.org/stash/dataset/doi:10.5061/dryad.54qt7	Cohort		✓(5)			✓ (8)	✓ (2)
Nichols et al. [59]	National Health Service (UK) Read Codes and British National Formulary (BNF) drug codes	15 to 24 years, representative of UK mixed ethnicity general population, to 2013	Primary	The Health Information Network database (THIN), a large dataset of anonymized electronic medical records extracted from general practices using Vision medical records software. National Health Service Read codes and British National Formulary drug codes	Not offered. Source identified	Cohort	✓ (6)	✓ (1)	✓ (5)		✓ (15)	✓ (2)
Półchłopek et al. [60]	ICPC P* code (psychological), T06* ICPC (anorexia, bulimia), 9 ATC values in N05–N07, 21 referral descriptions in Dutch	Electronic Medical Records (EMRs) from 76 general practices in the Leiden area, gathered, concatenated and preliminarily aggregated by a third party, Stichting Informatievoorziening voor Zorg en Onderzoek (STIZON3). 27% identified as having Mental Health Problem. Aged 0–19 from the period 2007–2017 (up to and including 31.12.2016)	Primary	Data sourced from the PIPPI project (‘‘Primary care integrated for identification of psychosocial problems in children’’ conducted in the Department of Public Health and Primary Care of Leiden University Medical Centre. Symptoms and diagnoses coded with International Classification of Primary Care (ICPC) standard (in Dutch); descriptive symptoms text mined from the notes of general practitioners (in Dutch); all GP encounters, including phone calls and visits; prescriptions coded with Anatomical Therapeutic Chemical (ATC) standard; measurements made by the GP or performed in a laboratory; referrals to specialists (in Dutch)	Not offered. Source identified	Cohort (with target/non target populations for disorders)	✓	✓			✓	
Qiu et al. [61]	Confirmed diagnosis of depression in 2016. Chronic Conditions Data Warehouse (CCW) algorithms by Centers for Medicare and Medicaid Services (CMS)	Subset of 7.2 million patients in a 3-year period, Patients enrolled between 2014 and 2016, with 2,099 variables including the diagnosis, procedure, medication, and health service provider information. Age < 65 years. Female ratio 56.48% (controls), 69.43% (cases). Mixed ethnicity. USA population	Primary, Secondary	MarketScan commercial claims and encounters database owned by IBM MarketScan R ©1 Research Database. 283 CCS (clinical classification software) codes, mapped from both ICD-9-CM and ICD-10-CM (Clinical Modification) codes in the MarketScan database. 242 CCS procedure codes, mapped from both ICD-10-PCS (Procedure Coding System) codes, Current Procedural Terminology (CPT) and Healthcare Common Procedure Coding System (HCPCS). Revenue codes, Place of service, Provider type, Service sub-category code, e.g. Magnetic resonance imaging (MRI), and positron emission tomography (PET) scans. 234 drugs and medications as defined in IBM Red Book	Not offered. Source identified	Case/Control		✓				✓
Sau and Bhakta [62]	Hospital Anxiety and Depression Scale (HADS)	520 geriatric patients attending hospital general Out Patients Department (OPD), Mean (± SD) age was 68.5 (± 4.85) years, 281 (55%) males and 229 (45%) females. Local population ethnicity	Primary, Secondary	Data source was the Kar Medical College and Hospital, Kolkata, West Bengal, India, data were collected from 520 geriatric patients attended at the general Out Patients Department of that hospital between January and August 2016. Storage format not specified, hospital data collection	Not offered. Source identified	Case/Control	✓ (6)	✓ (2)	✓ (1)		✓ (4)	
Souza Filho et al. [63]	Diagnostic and Statistical Manual of Mental Disorders-V (DSM V)	971 patients from 20 primary care units in the city of Rio de Janeiro. Mean age 57.67 (± 14.47). 64% male, 36% female	Primary	All data collected were included a posteriori by two blinded and independent researchers in an electronic clinical research form (CRF) database and was stored and managed using Research Electronic Data Capture (REDCap) hosted at Instituto Nacional de Cardiologia. All data were anonymized, as suggested in the General Data Protection Regulation	Not offered, source identified	Cohort	✓	✓				✓
Wang et al. [64]	ICD9/10 codes	EHRs from Weill Cornell Medicine and New York-Presbyterian Hospital from 2015 to 2017. Age 33.92 (SD 4.51) in non-PPD group; 34.36 (SD 4.61) in the PPD group. Ethnicities identified included White, Asian, American Indian or Alaska Nation, Black or African American	Primary, Secondary	All study data are represented using Observational Medical Outcomes Partnership (OMOP) common data model. All diagnoses were represented as Systematized Nomenclature of Medicine-Clinical Terms (SNOMED-CT) codes. Medication and dosage were standardized by Anatomical Therapeutic Chemical (ATC) Classification System	Not offered. Source identified	Case/Control	✓	✓		✓	✓	
Xu et al. [65]	70 ICD9/10 codes (45.7% ICD9 codes, 54.3% ICD10 codes) RxNorm (USA normalized naming system for generic and branded drugs) codes for drugs	11 275 patients with depression plus same number of controls from between January 2008 to November 2017. Age 18 to > 65. Mean age depressed 62.6 (SD 19.5). Mean age non depressed 63.7 (SD 20.1). Depressed cohort 69.0% female. Non depressed cohort 68.3% female. Race/Ethnicity included: White Black or African American Asian American Indian or Alaska Native, Native Hawaiian or Other Pacific Islander, Not Hispanic or Latino, Hispanic or Latino	Primary	INSIGHT Clinical Research Network (CRN) database. EHRs of 12 million patients from five large medical centers across New York City: Albert Einstein School of Medicine/Montefiore Medical Center, Columbia University and Weill Cornell Medicine/New York-Presbyterian Hospital, Icahn School of Medicine/Mount Sinai Health System, Clinical Director's Network, and New York University School of Medicine/ Langone Medical Center, 471 federally qualified health centers, safety net clinics, primary care practices, and hospice centers. Multiple comorbidities were also extracted based on the CMS Chronic Conditions Warehouse (CCW). Medication data was mapped to the Anatomical Therapeutic Chemical (ATC) Classification System	Not offered. Source identified	Case/Control	✓ (18)	✓ (4)		✓	✓ (17)	✓ (1)
Zhang et al. [66]	ICD-9 Codes	De-identified electronic health records (EHR) data from 10 schools participating in the College Health Surveillance Network (CHSN) from January 1, 2011 through December 31, 2014. The demography of enrolled students (sex, race/ethnicity, age, undergraduate/graduate status) closely matched the demography for the population of 108 Carnegie Research Universities/Very High classification	Primary	The selected 10 schools within the College Health Surveillance Network (CHSN) include 263,947 enrolled students representing all geographic regions of the United States. ICD-9 codes I extracted from primary care visits of 213,112 patients	Not offered. Source identified	Case/Control	✓ (2)			✓ (1)	✓ (2)	
ㅤ												

**Citation**	**Predictors** (Note 1)							**CEBM Level**	**Performance Metric**			
	**Social/Family**	**Somatic**	**Substance/Alcohol abuse**	**Visit frequency**	**Word list/text**	**Other measurements & predictors**	**Predictors considered (max)** Note 2	**Oxford Centre for Evidence Based Medicine for diagnosis (1 to 5)**	**Acc**	**Prec**	**Spec**	**Sens**
Abar et al. [49]	✓	✓	✓			✓	> 10,000	3	na	na	na	na
Geraci et al. [50]					✓		Note 3	4	na	0.77	0.68	0.94
Hochman et al. [51]			✓ (1)			✓ (2)	156	4	na	na	0.91	0.35
Huang et al. [52]				✓ (1)			> 1000	4	na	na	na	na
Jin et al. [53]		✓		✓			29	3	na	na	na	na
Kasthurirathne et al. [54]		✓		✓			1150	3	na	na	76.03—92.18	68.79—83.91
Koning et al. [55]	✓	✓		✓		✓	100 s	4	na	na	na	na
Meng et al. [56]		✓					> 1000	4	na	na	na	na
Meng et al. [57]		✓					> 1000	4	na	na	na	na
Nemesure et al. [58]	✓ (7)		✓ (5)			✓ (32)	59	3	na	na	0.66- 0.70	0.55—0.66
Nichols et al. [59]	✓ (15)	✓ (8)	✓ (2)	✓ (1)			60	3	na	na	na	na
Półchłopek et al. [60]		✓		✓		✓	3240	3	na	na	na	na
Qiu et al. [61]						✓	2099	3	na	na	na	na
Sau and Bhakta [62]	✓ (5)	✓ (5)	✓ (1)			✓ (1)	20	4	0.91	0.89	0.9	na
Souza Filho et al. [63]	✓	✓	✓			✓	34	3	0.89	na	na	0.9
Wang et al. [64]		✓				✓	98	3	na	na	0.391–0.616	0.867—0.959
Xu et al. [65]		✓ (5)	✓ (3)				500	3	na	0.61–0.89	na	0.58–0.91
Zhang et al. [67]		✓ (21)				✓ (2)	1000 s	3	0.56–0.58	na	0.40–0.50	0.60–0.70
ㅤ												

**Citation**	**Performance Metric**		**Baseline/Comparator**	**Range (Case / Controls) Training/Test (%/%)**	**Classifier (s)**	**Validation**	**Separate Holdout**	**Fitting**	**Code sharing and details**	**Ethical Approval**	**Citation**
	**F1**	**AUC ROC**									
Abar et al. [49]	na	na	Odds Ratio Lower Bound (ORLB)	> 3 million	Association rule mining (ARM)	None	No	Two-stage pipeline. Stage 1: Reducing the predictor codes into groups, e.g. 11,887 ICD 9 codes reduced to 282 classes. Stage 2: Identify rules then rank the 75,465 rules with depressive disorders and reduce to top 100. For top 100 novelty ratings were assigned on a scale of 1 to 5 (with 5 indicating most novelty) by a practicing psychiatrist. No statement on overfitting	Not stated. Algorithms discussed in main text. Software identified—Linear-time Closed item set Miner (Open Source Data Mining: Frequent Pattern Mining Implementations, OSDM '05. ACM; 2005. LCM Ver.3)	Not stated	Abar et al. [49]
Geraci et al. [50]	na	na	Performance	758 training/103 testing	Deep Learning (DL)	fivefold	Yes	Three-stage pipeline. Stage 1: EHR clinical notes data deidentified and features extracted using NLP. Stage 2: Creation of two DL models, one aimed at identifying those likely to develop depression and those not (to support patient selection for trials). Stage 3: Models combined to form composite model. No statement on overfitting, but hold out set used	Not stated. For deidentification Perl-based software package De-id V.1.1. For machine learning, R language implementation of the H2O.ai package, which includes a multilayer, feedforward deep neural network for the purpose of prediction under a supervised protocol. R programming language (wordnet, RKEA, tm, SDMTools)	Yes, Research Ethics Board-approved	Geraci et al. [50]
Hochman et al. [51]	na	0.71	AUC-ROC (Area Under Curve—Receiver Operating Characteristic)	185,029 (training split 80/20 for testing), 29,330 validation set	XGBoost (XGradient Boosting)	Yes	Yes	Two-stage pipeline. Stage 1: The main model was fitted using the full set of 156 predictors with initial validation using 20% of training set followed by separate testing on validation data set. A simpler model was also created based on questionnaire derived data. Performance was reported via AUC-ROC, bootstrapping was used to establish 95% confidence intervals. Stage 2: Shapley Additive Explanations (SHAP) was used to show impact of individual features in models. No statement on overfitting, but hold out set used	Not stated. R (R Foundation for Statistical Computing) version 3.4.3 (including the RMS and pROC packages) and Python 3.7.3 (Python Software Foundation)	Not stated	Hochman et al. [51]
Huang et al. [52]	na	0.70–0.80	AUC-ROC	5000 cases and 30,000 matched controls. (80% training, 20% test)	LASSO (Least Absolute Shrinkage and Selection Operator)	None	Yes	Two-stage pipeline. Stage 1: Terms used for defining depression case condition were excluded from the predictors prior to creating model using LASSO. Stage 2: The model is then validated on three test sets created for different cut off points: at time of diagnosis case date, and twelve months prior to that date. The output of the validation being ROC curves. No statement on overfitting, but hold out set used	Not stated. Least Absolute Shrinkage and Selection Operator (LASSO) logistic regression from the R glmnet package	Not stated	Huang et al. [52]
Jin et al. [53]	na	0.73–0.86	AUC-ROC	853 cases (80% training, 20% test)	Poisson Regression	None	Yes	Two-stage pipeline. Stage 1: 20 time varying factors and nine time-invariant factors relating to diabetes as predictors. Estimated effect of each candidate predictor as univariants and obtained p-values. Selected p < 0.05. Stage 2: Models evaluated at baseline, 6 months, 12 and 18 month follow up using ROC. No statement on overfitting, but hold out set used	Not stated. Fixed and random effects for a generalized multilevel model were estimated using quasi-likelihood estimation implemented by the “glmmPQL” function in R package “MASS”. Equations provided in text for generalized multilevel regression model, using the longitudinal dataset from a recent large-scale clinical trial	Not stated	Jin et al. [53]
Kasthurirathne et al. [54]	72–92 (apron)	0.78—0.94	AUC-ROC	84,317 patients. 90% training 10% testing	Random Forest (RF)	None	Yes	Two-stage pipeline. Stage 1: Using data extracted using NLP techniques and EHR ICD codes creating 5 data vectors (4 patient subgroups and 1 master data vector). Stage 2: These used to train RF models that were then applied to test set to derive AUCROC performance data. No statement on overfitting, but hold out set used	Not stated. Python programming language (version 2.7.6) for all data preprocessing tasks and the Python scikit-learn package for decision model development and testing	Not stated	Kasthurirathne et al. [54]
Koning et al. [55]	na	na	C-statistic (0.62–0.63), odds ratios	19,420 out of 70,000	Logistic Regression (LR), K-Nearest-Neighbours (KNN), Classification and Regression Tree (CART), AdaBoost (AB), Gradient Boosting (GB), Extreme Gradient Boosting (XGB), Random Forests (RF) and Support Vector Machine (SVM)	Bootstrap	No	Three-stage pipeline. Stage 1: Predictor variables derived from EHR data and those with low prevalence (< 1%)eliminated from data set. Data sets split by age group. Stage2: Logistic regression models trained on each age group dataset. Stage 3: The models were internally validated using bootstrap resampling (500 bootstrap samples) and estimating a shrinkage factor. Brier scores were calculated to assess the average prediction error. No statement on overfitting	Not stated. Analysis and modelling in SPSS (version 23) and R (version 3.5.1)	Yes, Ethics Committee of the Leiden University Medical Centre issued a waiver of consent (G16.018)	Koning et al. [55]
Meng et al. [56]	na	0.76 (PRAUC)	Comparison with RF model via PRAUC performance	10,148 (3,047 developed depression)	Multi-Level Embeddings of diagnoses, procedures, and medication codes with demographic information and Topic modelling (MLET)	None	No	Model trained on combined data set (including Breast cancer, Liver cirrhosis and MI). Results compared for prediction of depression at two weeks, three months, six months and one year prior to depression diagnosis. No statement on overfitting	Yes. The source code and more detailed description of the model is available at https://github.com/lanyexiaosa/brltm. BERT model was implemented in Pytorch 1.4. A visualization tool was identified: https://github.com/jessevig/bertviz	Yes. Patients for this work were identified from EHR in accordance with an Institutional Review Board (IRB) (#14–000204) approved protocol	Meng et al. [56]
Meng et al. [57]	na	0.77 to 0.81	Comparison with established models for varying times for prediction in advance of diagnosis (two weeks, three months, six months, one year). AUC-ROC and precision recall area under the curve (PRAUC)	10,148 (3,047 developed depression) 70% training, 10% validation, and 20% test	Hierarchical Clinical Embeddings combined with Topic modelling, LASSO, SVM, MLP, MiMe, RF, VAE + RF	tenfold	Yes	Three-stage pipeline. Stage 1: ICD codes (9,285, reduced by only using first three digits of code) used to identify features from EHR data followed by extraction of a further 100 features using Latent Dirichlet allocation (LDA) for pre processing clinical notes. Stage 2: Models fitted to EHR/Clinical Notes data. Models created for depression and also for prediction of three comorbidities, breast cancer, liver cirrhosis and myocardial infarction. Stage 3: Models were assessed for predictive value at two weeks, three months, six months and one year prior to case incidence. No statement on overfitting, but hold out set used	Yes. All models created in TensorFlow 1.12. Equations for models provided in text. The source code of HCET is available at https://github.com/lanyexiaosa/hcet	Yes. Patients for this work were (as per Meng et al. [56]) identified from EHRs in accordance with an Institutional Review Board (IRB) (#14–000204) approved protocol	Meng et al. [57]
Nemesure et al. [58]	na	0.67–0.73	AUC-ROC	4184, 70% training (N = 2929) and 30% (N = 1255) held out testing	XGBoost, Random Forest, Support Vector Machine, K-nearest-neighbours and a neural network with Bayesian fine tuning, logistic regression	fivefold	Yes	Two-stage pipeline. Stage 1: Predictions from each classifier are generated using fivefold training on the training data, Stage 2: Predictions from all models are used to train XGBoost classifier on the test data, which consists of predictions made by the six classifiers. No statement on overfitting, but hold out set used	Yes, Code written in python and used sklearn. Vignettes available at https://github.com/mnemesure/MDD_GAD_EHR. Imputation for missing values using a Bayesian Ridge approach. SHAP (Shapley Additive Explanations) scores were utilized calculate and visualize feature importance this complex model	Yes, National Data Protection Authority (NCIL) approved the original study from which data was sourced. This study received institutional exemption from the Committee for the Protection of Human Subjects at Dartmouth College	Nemesure et al. [58]
Nichols et al. [59]	na	0.70 -0.72	AUC-ROC	98,562 cases and 281,248 matched controls, 70% training, 30% test	Backward stepwise conditional logistic regression	None	Yes	Prediction from the logistic regression to generate ROC curves using test data. No astatement on overfitting but hold out set used	Not stated. STATA was used for statistical analyses and to implement ML models	Yes, Scientific Review Committee on 3 Oct 2014 (SRC Ref: 14–056)	Nichols et al. [59]
Półchłopek et al. [60]	na	0.582 to 0.782	AUC-ROC	92 621 (27% case positive, 63% controls) split by age group (70% for training, 30% test)	Logistic regression, SVM, regression tree, random forest, deep neural network and XGBoost	threefold	Yes	Two-stage pipeline. Stage 1: Patients with insufficient medical history were excluded and case positive patients had medical history excluded after the event and within a fixed time window before it. Then divided into 5 age groups (0–3, 4–7, 8–11, 12–15, 16 +). Stage 2: Models training using training subset and performance evaluated using the test set. For the best performing classifier, XGBoost, variable importance data was calculated. No statement on overfitting, but hold out set used	Yes, Code algorithms and sample code provide in appendices, mathematical basis provided in main text	Not stated	Półchłopek et al. [60]
Qiu et al. [61]	na	0.75–0.76	Prediction vs clinical outcome	Case = 254,648/ control = 6,969,972 (training 75%, testing 25%)	Least Absolute Shrinkage and Selection Operator (LASSO) and Random Forest (RF)	None	Yes	Two models were created the first using a form of penalized regression (LASSO) the second using a decision tree based method (RF). AUCROC was calculated for the models and odds ratios were derived. No statement on overfitting, but hold out set used	Not stated. Details of, e.g., regularization and depth parameter definitions in main text	Not Stated	Qiu et al. [61]
Sau and Bhakta [62]	na	na	Performance vs. HADS—independently assessed	48.2% case and 51.8% healthy controls, 520 training set (83%) and 110 test set (17%)	Random Forest, Bayesian Network, Naïve Bayes, Logistic, multiple layer perceptron (MLP), Naïve Bayes (NB), random forest (RF), random tree (RT), J48, sequential minimal optimisation (SMO), Random sub-space (RS), and K Star (KS)	tenfold	Yes	Two-stage pipeline. Stage 1: The initial classifiers were subjected to feature selection approaches using machine learning technology in Waikato Environment for Knowledge Analysis (WEKA). Stage 2: Training and testing was done using a ten-fold cross validation method and the classifier with the highest predictive accuracy was then validated against the external (110 instances) data set. No statement on overfitting, but hold out set used	Not stated. Coding system specified, Waikato Environment for Knowledge Analysis (WEKA) (version 3.8.0) (http://www.cs.waikato.ac.nz/ml/weka/documentation.html). Main text in paper describes procedures used	Yes. Ethical clearance from the Institutional Ethics Committee of R.G. Kar Medical College and Hospital, Kolkata, West Bengal, India. Informed consent was taken from every patient before data collection	Sau and Bhakta [62]
Souza Filho et al. [63]	na	0.87	AUC-ROC	971 patients (881 non-depressive and 90 with depression)	Logistic Regression (LR), K-Nearest-Neighbours (KNN), Classification and Regression Tree (CART), AdaBoost (AB), Gradient Boosting (GB), Extreme Gradient Boosting (XGB), Random Forests (RF) and Support Vector Machine (SVM)	tenfold	No	Two-stage pipeline. Stage 1: Synthetic Minority Oversampling Technique (SMOTE) was used to resolve imbalances in the data set. Stage 2: The models were built and cross validation used to determine performance (AUCROC). No statement on overfitting	Not stated. “R” statistical software to perform the randomization for trial. Machine learning implemented in the Python 3 programming language	Yes. The study protocol was approved and monitored by Instituto Nacional de Cardiologia in Brazil. All patients signed informed written consent	Souza Filho et al. [63]
Wang et al. [64]	na	0.69—0.79	AUC-ROC	9980 (769 cases, 9211 controls)	L2-regularized Logistic Regression, Support Vector Machine, Decision Tree, Naïve Bayes, XGBoost, and Random forest	tenfold	No	Two-stage pipeline. Stage 1: To down select predictors univariate logistic regression (LR) analyses select those with p-values below 0.05. Stage 2: models were built using the different classifiers and performance measured as AUCROC by validation, Additionally Odds Ratios and variable importance were established to provide interpretable data. No statement on overfitting	Not stated. All machine learning and statistical analyses were performed with R version 3.4.3	Not stated	Wang et al. [64]
Xu et al. [65]	na	0.80—087	AUC-ROC	11,275 case /11275 control	Logistic Regression (Ridge), Support Vector Machine (SVM), Random Forest (RF), Gradient Boosting Decision Tree (GBDT)	fivefold	No	Two-stage pipeline. Stage 1: Identify 500 features for participants based on selection criteria. Stage 2: Train models and generate performance data. Generate Heatmaps using Clustergrammer. No statement on overfitting	Not stated. For Ridge, RF, SVM, used Scikit-learn software library, for the GBDT, used XGBoost software library, both Python based	Not stated	Xu et al. [65]
Zhang et al. [66]	na	na	Comparison of frequency, pairwise, and M-SEQ representations methods	7322 case/ 205,790 control	SVM, LDA, and RF for models based on frequency, pairwise, and M-SEQ representations	fivefold	No	Two-stage pipeline. Stage 1: To avoid imbalance issues from unmatched case/control ratio EasyEnsemble used to prevent the majority class from dominating the learning process. Stage 2: frequency, pairwise, and M-SEQ models used to create SVM, LDA, and RF models. No statement on overfitting	Not stated. Equations and algorithms included and described in text. Software not specified	Not stated, work supported by College Health Surveillance Project	Zhang et al. [66]
									Not stated. STATA 14 software was used for statistical analyses but it is not clear if this was used to implement ML	Yes. Institutional Review Board at Weill Cornell Medicine (IRB protocol# 1,711,018,789)	Zhang et al. [67]

Note 1: The predictor categories are further described in main text (results section). Where it was practical to obtain/estimate numbers in brackets have been given for the predictor count within the category for models—these are indicative only, especially where multiple models were created

Note 2: The total number of predictors used was difficult to determine at a summary level as multiple models used different combinations, in some cases no exact number was provided but a reference to a set of definitions used as a starting point. The number given in the table is the maximum used either as stated or estimated

Note 3: For Geraci et al. [50] the number of predictors/features extracted from EHR text entries is not defined. No estimate has been made

Note 4: In the paper by Półchłopek et al. [60], the use of "*" after the ICPC code, e.g., T06*, indicates all codes under that heading

### Depression definition

The definition of depression and the method of its classification varied across the studies in this review. A combination of depression diagnosis definitions based on NHS Read codes [68], SNOMED (Systematized Nomenclature For Medicine) [29] codes, ICD [12] or DSM [13] based assessments and/or the prescription of antidepressants (ADs) was used in 16 of the 19 studies. Only one study, by Xu et al. [65], used antidepressant prescription alone as a case definition. Three other studies relied on the use of a validated questionnaire such as the PHQ-9 [69] or HADS [16].

### Predictors

Here we report on aspects of the predictors including their definition, how we grouped them and their frequency of use.

#### Definitions

Most predictors were derived from a combination of variables present in the EHR databases (e.g., SNOMED/NHS Read codes and/or prescription of a drug in a similar way to the definition used for depression) and were typically categorical. In some cases, additional parameters specifying a time frame for the predictor were also available. Some predictors were defined by identifying components by pre-processing clinical notes/other textual information. A few studies used non categorical predictors such as physiological measurements for example Body Mass Index (BMI), blood pressure, and cholesterol as predictors. This was usually where participants were receiving some form of secondary care, such as in pregnancy for PPD prediction.

### Groups

No formal method for grouping predictors was evident in the studies and, due to the large number of diverse predictors used in different papers, for clarity these were organised into the following groups. Specifically: comorbidity, demographic, family history, other (e.g., blood pressure), psychiatric, smoking, social/family, somatic, obstetric specific, substance/alcohol abuse, visit frequency and word list/text. Due to this flexibility in definition, there are overlaps between studies concerning which category a predictor might fall, for example a blood test may be in “other, or “obstetric specific”. Table 5 shows the predictors groups and commentary on their content.

**Table 5:** Grouping of predictors from the studies

*Predictor group*	*Commentary*
Comorbidities	Comorbidities were included in 13 studies. They included long-term conditions, such as diabetes, asthma, epilepsy, and chronic pain. These were commonly used, especially when the study authors highlighted theoretical links with depression
Demographic	Demographic predictors were used in 16 studies. On some occasions, specific demographic variables were excluded due to insufficient availability/coverage (often the case for ethnicity). Gender was included as a predictor and occasionally also as a means of creating gender-specific models (e.g., Nichols et al. [59]). Social deprivation was also used as a predictor, and information about missed immunization(s) was used in two studies, Nemesure et al. [58] and Nichols et al. [59], as a proxy for social deprivation The age range of cases was often an integral part of the study’s specific aims. Age being treated either as a numeric or to break up the study population into subgroups. Some studies specifically focussed on older patients. For instance, Sau and Bhakta [62] used data with an average age of 68.5 years (standard deviation 4.85 years), whereas Nichols et al. [59] focused on early diagnosis among young people, between 15 to 24 years of age. Some studies narrowed the analysis to a narrow age bracket, others included a wide range of ages. For example, Hochman et al. [51], who studied postpartum depression reported an average age of 29.4 years (standard deviation, 5.4) whereas Xu et al. [65] used data from participants whose age ranged from 18 to over 65
Family History	Family history was used in five studies and included family history of abuse (physical/sexual) and drug/substance abuse, often because the study authors cited theoretical links with depression. This group of predictors was often under recorded, as reported in the Nichols et al. [59] study where family history data was removed from the model due to low prevalence (< 0.02%) in their data. Insufficient family history data was also highlighted as a limitation in other studies [53, 55]
Obstetric specific	Obstetric specific were used in five studies focussed on the prediction of postpartum depression, and these included predictors such as premature birth, use of specific drugs during pregnancy and obesity. This type of predictor was also used in non-postpartum depression studies e.g., Abar et al. [49]
Psychiatric symptoms or other diagnoses	Psychiatric symptoms/diagnoses were used in fifteen studies. These include both depression related symptoms such as: anxiety, low mood, self-harm, sleeping and eating disorders, too little sleep etc. They also include the broader range of conditions including post-traumatic stress syndrome, obsessive compulsive disorder, personality disorders and psychoses. Within individual studies there may/may not be a distinction made between these two subgroups
Smoking	Smoking was used in seven studies. However, it was identified, for instance by Nichols et al. [59], that data may be incomplete for all participants and that this might impact the ability to reliably assess correlations with depression, to mitigate this they used “missing smoker” data as a separate predictor. This was a categorical predictor in the selected studies
Social/family	Social and family related factors were used in seven studies these included bereavement, divorce, single parent, police or social services involvement and similar
Somatic	Somatic conditions were used in 14 studies these include physical conditions such as, abdominal pain, back pain, dyspepsia, eczema, headaches, and others
Substance/alcohol abuse	Alcohol/substance abuse was used in seven studies, participants identified as having drug/alcohol abuse problems. Typically categorical, but some studies included levels of abuse and/or combinations of the two
Visit frequency	Visit frequency was used in six studies and shown to be a significant contributor to model performance. This is an integer variable based on number of visits in a specified period to the primary care facility (e.g., NHS GP)
Word list/text	Word list/text derived data was used in only one study, Geraci et al. [50], this was a source of data that was then analysed, using natural language processing, to extract predictors from clinical notes. It is based on language/defined terms specific
Other measurements and predictors	Other measurements and predictors were used in 11 studies and included, e.g., measurements of physical characteristics such as blood pressure, cholesterol, results of assays, and height/weight

Note: There may be overlap or gaps in these groupings as the predictors used and the reason for their use is study specific and not always explained

**Fig. 2:**
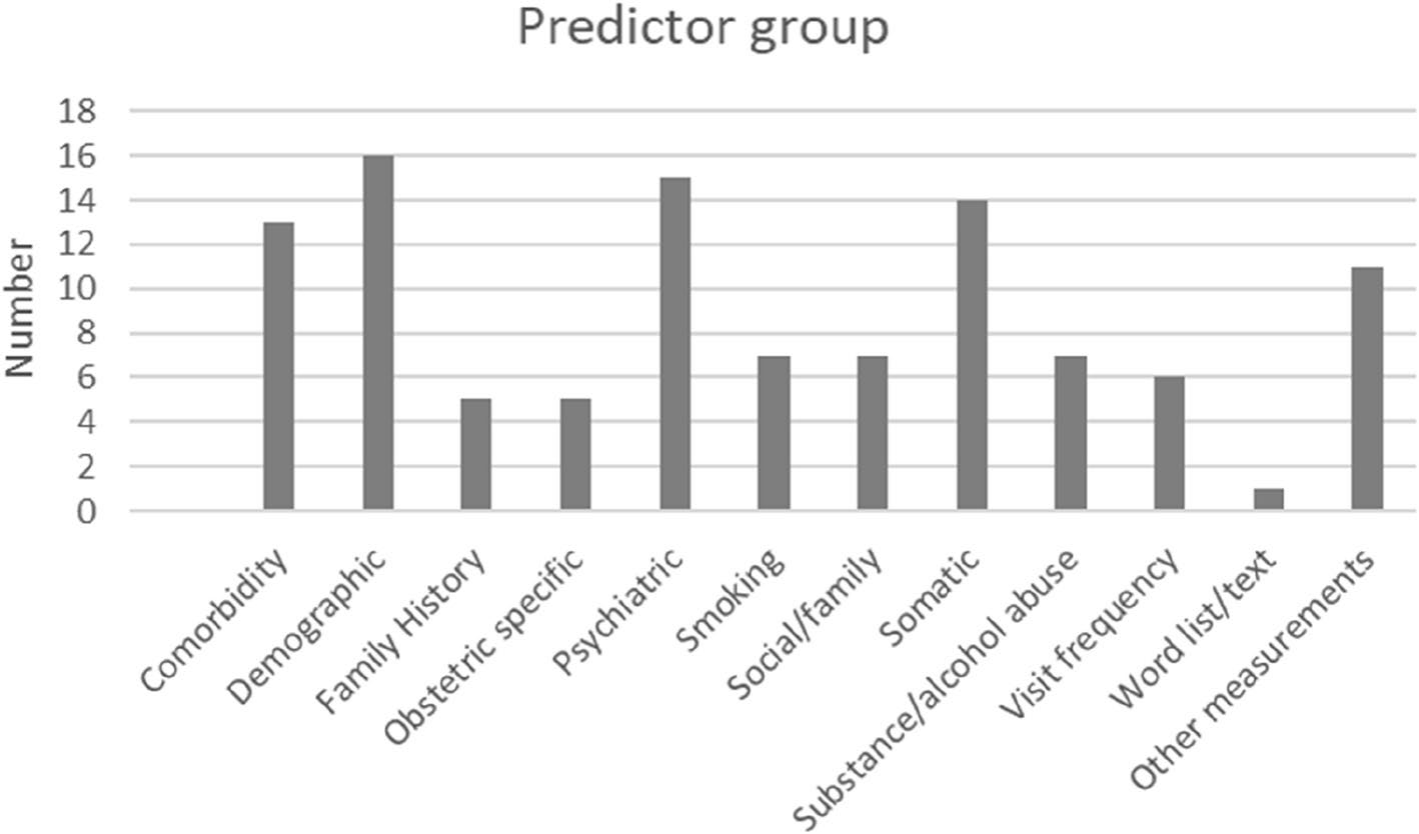
The approximate number of studies using different groups of predictors. Note 1: Some papers used multiple categories of predictors and not all categorised them. Note 2: The total number of predictors used was difficult to determine at a summary level as multiple models used different combinations, in some cases no exact number was provided but a reference to a set of definitions used as a starting point

Figure 2 indicates frequency of predictor use across the selected studies.

### Data

The studies in this review used data sets from EHRs systems, insurance claims databases and health service (primary and secondary) providers. As such they store, organise, and define data in a variety of ways that are not expected to be consistent with each other. Most of this data is categorical in nature, though some predictors such as blood pressure, are usually continuous variables within a range. In this section we report how each of the reported studies dealt with missing or erroneous data, potential sources of bias. We also report whether the authors made their data and/or code publicly available.

### Missing or erroneous data

Missing data either related to missing patients and/or missing predictor data. In both cases it may not be possible to know that the data is missing. For missing patients, Koning et al. [55] excluded patients whose records did not identify gender or had no postcode registered. Huang et al. [52] removed entries where patients had less than 1.5 years of visit history. Wang et al. [64] excluded from the analysis PPD patients for whom there was no third trimester data.

With regard to missing data. Nemesure et al. [58] estimated that, for their data set, missing values were present in 5% of the data overall and for 20 out of the 59 predictors they used. In some studies, missing data led to exclusion of cases from the analysis. In Nichols et al. [59]. missing smoking status was used to infer non-smoking on the basis this was less likely to be missed for smokers/those with smoking related disorders. Missing data also led to exclusion of predictors. Again, in Nichols et al. [59], the authors did not use ethnicity as it was missing in over 63% of patients. Similarly, Zhang et al. [67] excluded ethnicity from their USA dataset for the same reasons. Many studies (e.g., Koning et al [55]., Meng et al. [57], Nichols et al. [59] raised concerns that errors in predictor data could affect performance, generalizability, and reliability of the models. Errors and missing data were identified as being due to misclassification, measurement errors, data entry and bias; all of which can be difficult identify and/or correct in EHR data as noted by Wu et al. [36]. Other studies varied in the strategies used for dealing with missing data. Common approaches were to estimate the level for a missing point or simply acknowledge that remedial action was not available. Nemesure et al. [58] used an imputation approach fortheir numerical data, such as blood pressure. Where remedial action is not possible then the patient might be excluded from the study, e.g. Hochman et al. [51].

### Sources of bias

Many of the studies (12), for instance, Hochman et al. [51], Huang et al. [52] and Koning et al. [55] raised the question about data bias due to cohort selection or collection processes, such as diagnosis, data interpretation and system input. Other studies (12) recognised sources of bias impacting accuracy and generalizability. Jin et al. [53] identified that as the population in their study were mainly Hispanic and there was incompleteness of comorbidity predictor data (e.g., for diabetes), both performance and generalizability would be affected. Zhang et al. [67] acknowledged that sourcing their data from an urban academic medical centre could introduce result in a limited generalizability of their findings. Hochman et al. [51] suggested that their use of an exclusion criteria removing severely depressed patients based on the prescription of specific drugs could also create bias. Zhang et al. [66] chose to exclude ethnicity from their models due to coding inconsistencies and errors; making a bias in that area a potential issue. Huang et al. [52], defined depression based solely on antidepressant usage and suggested their sample would be skewed towards the more severely depressed because the sample excluded those whose condition was treated with only psychotherapy or those without any treatment. A similar concern regarding changing definitions for the detection of depression during their study period was expressed by Xu et al. [65]. At a broader level, 20 of the studies were from “WEIRD” (Western, Educated, Industrialised, Rich, Democratic) countries with the majority (15) from the USA. The remainder were from countries with highly developed IT and healthcare industries such as Brazil, Israel, and India.

### Data sharing

The nature of the data, data protection and requirements for anonymity, and privacy issues limited access to source data though details of sources themselves were more often made available (e.g., Hochman et al. [51], Nichols et al. [59]).

### Modelling

**Fig. 3:**
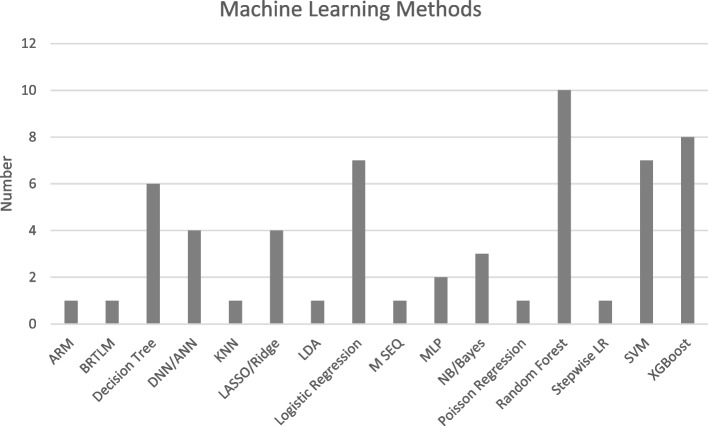
Machine Learning/Artificial Intelligence Methods for pre-processing and modelling (note LR variants add up to 11). Abbreviations:; ARM, Association Rule Mining; BRTLM, Bidirectional Representation Learning model with a Transformer architecture on Multimodal EHR; DNN/ANN, Deep Neural Network/Artificial Neural Network; KNN, K Nearest Neighbours; LASSO, Least Absolute Shrinkage Selection Operator; LR, Logistic Regression; MLP, Multilayer Perceptron; M SEQ, multiple-input multiple-output Sequence; NB, Naïve Bayes; SVM, Support Vector Machine; XGBoost, eXtreme Gradient Boosting

In this review, we identified a wide array of statistical techniques used on EHR data (see Table 4). Many different types of supervised ML were used for classification of depression versus control, including regression models (13 studies) and Random Forest (8 studies), XGBoost (8 studies) and SVM (7 studies) were the most common techniques. Use of multiple techniques in a single paper was also common, for instance Xu et al. [65] and Zhang et al. [66] used four or more methods. Geraci et al. [50] was the only study to use a deep neural network-based deep learning approach as the primary component of their model. Figure 3 summarises methods used in the selected studies.

Temporal sequence was referred to in two studies [49, 60] though other studies refer to time between predictors and diagnosis (e.g., Meng et al. [56]). In other studies patterns of predictors were used to determine their predictive probabilities of depression, sometimes using time constraints, such as a primary care visit “within the last twelve months” or specifically including time distant events such as birth trauma (Koning et al. [55], Nichols et al. [59]. Only one study, Półchłopek et al. [60], implemented temporal sequence, whereby the order of presentation of symptoms was considered, in the EHRs. Though Abar et al. [49] speculated that temporal sequence might be used to improve performance by taking causal sequence into consideration.

Most studies (17 out of 19) validated their models, most commonly (12) by splitting data into a training and a testing set. Cross validation data sets for model testing were also used (11 out of 19). Generally testing and validation was carried out by the same team as created the models, only Sau and Bhakta [62] had diagnostic accuracy checked by an independent team. Only one study used a separate data set for testing rather than splitting the original data set, Zhang et al. [67].

### Code sharing

Code was made available by the majority (12) of studies. In some cases, just the details of the packages that implemented the ML algorithm were provided. For example, Jin et al. [53] reference the R package MASS, rather than the providing the complete code.

### Performance

Several performance metrics was used to evaluate ML models of depression. Among those, researchers reported confusion matrices; area under the curve – receiver operating characteristics (AUC-ROC); and Odds Ratios/Variable Importance for predictors.

Confusion Matrix derived metrics (True Positives, True Negatives, False Positives and False Negatives) were used in sixteen of the studies, usually in conjunction with other measures particularly AUC-ROC. Many performance metrics are derived from this information, including accuracy, F1, sensitivity, specificity, and precision. Sensitivity (also known as recall) and specificity were commonly reported, possibly because they give information relating to the discriminative performance of the model and are well understood by practitioners [70].

For sensitivity, reported values range from 0.35 Hochmam et al. [51] to 0.94 Geraci et al. [50]. For specificity, reported values range from 0.39 Wang et al. [64] to 0.91 Hochman et al. [51]. Sensitivity was usually higher than specificity across the models with the exceptions being: Hochman et al. [51] who reported a high specificity figure of 0.91 with a low sensitivity of 0.35 using a gradient boosted decision tree algorithm; and Nemesure et al. [58] reported specificity of 0.7 and sensitivity of 0.55. The highest accuracy at 0.91 was reported by Sau and Bhakta [62] and the lowest was 0.56 (Zhang et al. [67]). This metric only gives a broad overall picture of correctly predicted results vs. all predictions made and gives no indication of the more useful true/false positive rates; it was presented in only six studies.

**Fig. 4:**
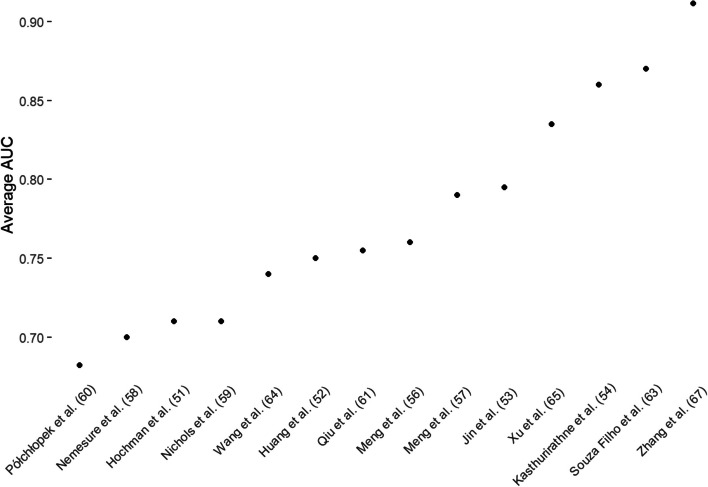
Average AUC performance across studies reporting them (AUC average = 0.78, Standard Deviation AUC Average = 0.07)

For the studies that reported performance in terms of AUC- ROC metric (14) the low extreme for any model was 0.55, specifically from a benchmark model predicting depression in the 12–15 years age group (Półchłopek et al. [60]. The highest AUC-ROC score was 0.94 (Zhang et al. [67], Kasthurirathne et al. [71]). The overall range AUC-ROC values reported was 0.70 to 0.90. The average AUC-ROC value was 0.78 with a standard deviation of 0.07. Figure 4 shows the average AUC values achieved in each study.

### Generalizability and interpretability

Generalizability was mentioned in 14 studies, for example Jin et al. [53] and Zhang et al. [67]. The points already illustrated under, “sources of bias”, for example, demographically specific participants, and, factors relating to missing data and granularity of data, such as only having social deprivation data at practice level have negative consequences for generalizability.

Interpretability was identified as a concern in only 3 studies (Koning et al. [55], Nemesure et al. [58], Meng et al. [56]). For interpretability Nemesure et al. [58] used SHAP (Shapley Additive Explanations) scores which offers a decision chart and other visualisations for model predictors [72]. None of the included studies provided visualisations other than AUC-ROC diagrams and bar charts, as such interpretability was not significantly addressed in the selected studies.

### Quality of studies

All the included studies achieved a score of 3 (11) or 4 (8) based on the OCEBM criteria (1 to 5 from highest to lowest) hierarchy of levels of evidence as far they could be applied to the selected studies, areas that related to diagnostic tests only (no interventions). This represents a moderate level of performance. Overall, the studies represented large sample sizes, usually case series or cohort trials and they applied a clinically recognised benchmark, had there been randomized trials studies could have been promoted to level 2.

Only 3 studies provided reference to the use of a formal assessment method such as TRIPOD [42]. suggesting that following standards is not yet widespread or that the frameworks are not yet sufficiently established or appropriate. This lack of consistent reporting is a limitation, and the use of standardised frameworks should become the expectation rather than the exception.

## Discussion

In this review we have identified three areas of interest: generalizability (can the model be reused with, e.g., different populations), interpretability (is the model’s information readily understandable to its users), and performance (does the model meet the needs e.g. in AUC-ROC, for the purpose for which it is intended) as key components to consider for predictive models of depression built on the use of ML with EHR data. All three would need careful evaluation before moving from research to a clinical application environment.

### Generalizability

This is a significant consideration for medical ML applications, whilst a model may work well in their development and testing environments, this does not guarantees that they will work in a new context [73, 74]. To be widely deployed clinically, the models in the studies would need to be generalizable, i.e., be able to work reliably outside of their development environment. Kelly et al. [73] identified the ability to deal with new populations as one prerequisite for clinical success. Areas identified in the studies that could impact generalizability included demographics, sources of bias, inclusion/exclusion criteria, missing/incomplete data, the definition of depression and predictors. All of these were identified in the included studies, for instance, Jin et al. [53] identified Hispanic participants being highly represented in their data and Zhang et al. [66] excluding ethnicity from their models.

As noted in the Performance sub-section of the Results, the ML method itself did not seem to be overly critical for outcome performance using the EHR data sets in the included studies and it is provisionally suggested that the method itself may be more generalizable than the data to which it is fitted.

Another area that can limit generalizability is the wide variety of EHR data. This varies depending on source for example insurance derived, a state health service such as the NHS, or a proprietary standard such as SNOMED etc. The coding may, or may not, incorporate a recognised medical standard such as the ICD [12] or DSM [13] amongst others that can be found in the included studies. Although not derived from the studies directly it was noted that individual EHRs systems are proprietary in nature and there is no universally accepted extant standard detailing how data should be categorised, stored, and organised for them.. There are organisations developing, promoting, and gaining accreditation, for example Health Level Seven International [75] with ANSI (American National Standards Institute) [76]. However, none of these are globally adopted, and the only accepted standard developed by the World Health Organization (E1384) was withdrawn in 2017 [77]. Lack of standardisation is currently a barrier to portability for individual applications. Consequently, it is likely that models are data source specific to a greater or lesser extent. Further work needs to consider how this can be addressed.

The studies in this review differed in how depression was defined and by the range of predictors selected and their definitions. As mentioned, a commonly used approach was to use a combination of EHR data entry codes covering diagnoses in combination with prescription of an antidepressant. This can result in too many cases as being diagnosed as depressed due to antidepressants being used for a wider range of conditions. Similar issues apply for the definition of predictors. In combination this restricts the generalizability of any models produced.

Another factor for generalization is the robustness of the models and their replicability. None of the studies included replication of their results, only Sau and Bhakta [62] used an independent team for the verification of results, though the majority employed recognised validation techniques and 12 used separate hold out data set. This last point is also relevant to establishing if models have been overfitted to their data; the possibility for this was not reported in any of the studies despite being known as a serious potential issue for ML models in general. Reducing bias and independent validation and testing is recommended for future work involving the prediction of depression using ML with EHRs.

### Interpretability

Interpretability was only identified as a concern in a few studies. However, clinical practitioners may wish to know the explanation for ML algorithm’s predicted diagnosis so they can fit it into a broader diagnostic picture rather than treating it as a “black box” as described by Cadario et al. [78]. Similarly, Vellido [79] and Stiglic et al. [80] also considered that interpretability and visualisation are important for effective implementation of medical ML applications. This may be as simple as listing the specific predictors that contributed to the outcome, for example, anxiety, low mood, chronic pain or similar. Of the included studies Nemesure et al. [58] used SHAP (Shapley Additive Explanations) scores which have been used in clinical applications [81] to aid interpretability, again by identifying the most important predictors. Techniques such as SHAP, and e.g., LIME (Local interpretable model-agnostic explanations) [82] offer visualisations which may be more intuitive and provide more easily digested information. However, none of the other studies included provided visualisations other than AUC-ROC diagrams and bar charts of predictors. That said, there is a long-standing unsettled debate regarding interpretability going back to the 1950s. Providing interpretive data to support a practitioner as opposed to a “black box” approach where the diagnosis made by the application is simply accepted, can lead to a lower diagnostic performance overall [83, 84]. It is recommended that future studies should be made that not only develop predictive models but also include trialling their use, for example with primary practitioners, support staff and/or patients, offering different forms of interpretable/black box output and assessing acceptability. This needs not be done, initially, in a clinical setting, but can be piloted and demonstrated in prototype form in a controlled environment. This can then be assessed using a combination of qualitative and quantitative methods e.g., with surveys, interviews, focus groups and panels prior to moving to clinical trials.

### Performance

Here we consider what may be limiting the performance of the models with respect to their intended used as a means of identifying depression. One limiting factor on performance in the included studies, relates to the definition of depression itself and the predictors used. Defining depression accurately is critical as this definition is used to train the ML application, a point raised by Meng et al. [57]. In the studies reviewed here, typically a combination of diagnostic and drug codes within the EHRs were used. Using prescription of antidepressants as part of the definition may misidentify too many cases, a point identified in the selected studies by, for example, Qiu et al. [61] and Nichols et al. [59]. ADs are prescribed for other conditions including anxiety [85, 86], chronic pain [87, 88], obsessive compulsive disorder [89, 90], post-traumatic stress disorder [91, 92] and inflammatory bowel disease [93]. Of the included papers Xu et al. [65] suggested that under-identification of depression cases could also occur for patients receiving treatment via private care or an alternate service provider.

The prevalence of predictors can be artificially boosted, as suggested by Koning et al. [55] and Nichols et al. [59] where primary care physicians who think a patient has depression may identify or suspect a precursor or comorbidity, for example, with other mental health conditions like low mood or anxiety. There is strong evidence that family history of depression, alcohol, drug, physical and sexual abuse, and co-morbidity with other mental health conditions, are strong predictors of depression [94–97]. However, this data appears to be under recorded resulting in removal of important predictors due to low prevalence—again in Nichols et al. [59] removed family history data due to its low prevalence (< 0.02%). This would be expected to have a negative impact on performance. Identifying consistent and valid definitions for depression and any predictors used is a necessity.

The studies in this review reported an overall model performance where AUC-ROC value was 0.78 with a standard deviation of 0.07 (Fig. 2). This compares well with primary care where up to half of depression cases are missed at baseline consultation, improving to around two thirds being diagnosed at follow up [38, 40]. An earlier paper by Sartorius et al. [98] reported that only 39.1% of cases of ICD10 current depression were identified by primary care practitioners. Based on the studies we identified potential areas that might support improvements in the performance of the models. A key area relating to this is that of over/under diagnosis; as mentioned in our background section early diagnosis and thus intervention can show benefits for depression [25, 99]. However, there is a broader argument with regard to over-diagnosis (i.e., false positives) in terms of potentially wasting resource or stigmatising patients.

Although some studies suggested that using more sophisticated techniques should improve performance, we noted that simpler methods such as logistic regression were often comparable to those obtained using more complex ones such as Random Forest and XG Boost (e.g., Zhang et al. [67]. Christodoulou et al. [100] echoed this conclusion in their systematic review of clinical prediction using ML where they saw similar performance for logistic regression compared with ML models such as, artificial neural networks, decision trees, Random Forest, and support vector machines (SVM). Geraci et al. [50] employed a deep neural network (deep learning) as their main modelling technique and Nemesure et al. [58] used it as a component in a larger ensemble model. However, neither demonstrated performance benefits from its use. Even if higher performance could be obtained using deep learning it is important to note that small amounts of noise or small errors in the data can cause significant reliability issues due to misclassification due to very small perturbations in the data [101, 102]. The use of more sophisticated techniques to improve performance is not supported by this review.

How else might performance be improved? The use of non-anonymised data, sourced from within a primary or secondary care facility, something that is more achievable in a clinical than a research setting, could be beneficial. For example, in the Nichols et al. [59] study social deprivation indices were only available at a regional/practice level and inspection of their model suggests that social deprivation has little impact on prediction of depression. This is inconsistent with expectation, as supported by Ridley et al. [103] who showed that there is a link between increased social deprivation and the probability of developing depression. Having this data at an individual level might be expected to increase the performance of a model. However, this is likely to only be achievable in a clinical trial of an application. Alternatively, the use of synthetically generated EHR data [104, 105] removes the patient confidentiality and related ethical constraints that come with real data and would allow all aspects of a model to be fully evaluated as if with non-anonymous patient data.

Another approach is using more information relating to time in predictive models; EHRs typically time stamp entries so it is known when a predictor is activated. Półchłopek et al. [60], considered temporal sequence in EHRs. They were concerned that techniques including support vector machines and random forest identify predictors that affect the outcome but do not identify the effect of sequence on that outcome. They looked at the improvement that could be found by using temporal patterns in addition to non-time specific predictors and noted a small positive effect. Abar et al. [49] also speculated that temporal sequence might be used to improve model performance. There are techniques that might be used to do this. For example, time series analysis methods such as Gaussian processes, which are capable of coping with the sparse nature of EHR data [106] have been used to make predictions for patients with heart conditions. We recommend exploring the use of more time dependent factors in building predictive ML models for depression.

Although missing data is more of a concern in terms of generalizability, some studies identified it as an opportunity to improve performance. Kasthurirathne et al. [54] noted that missing EHR data can reduce model performance and suggested that this could be mitigated by merging with other data sources, for example, related insurance claims. Nichols et al. [59] used missing smoking data as a predictor and it had a positive effect in their model. Missing data is potentially of significance of itself and is an opportunity for further study.

### Strengths and limitations

As far as we are aware this is the first systematic review focussed on the use of EHRs to predict depression using ML methods. The choice of journal databases and thedate range covered by the searches means that the studies identified provide a sound basis for comparison. The data extraction protocol was informed by established standards [42–44] to best identify data needed to support meaningful and repeatable analyses.

A limitation of this study is that inclusion criteria focused on study titles and key words which may have led to some ML studies using EHRs being missed. This was mitigated using backwards and forwards citation searches. Additionally, the variety of study designs including case control, cohort, and longitudinal studies precluded the possibility of using some of the more traditional quality assessment tools; we did however, as stated in methods, use OCEBM which has been used in previous ML systematic reviews. The categorization, definition, and identification of the numbers of predictors used within models was sometimes difficult to establish, leading to limitation in the scope of this information presented. It is also likely that the included studies are culturally specific as they focused on “WEIRD” populations.

## Conclusions

In conducting this systematic review, we have shown that there is a body of work that supports the potential use of ML techniques with EHRs for the prediction of depression. This approach can deliver performance that is comparable to, or better than that found in primary care. It is clear there is scope for improvement both in terms of adoption of standards for both conducting and reporting the research and the data itself. The development of an acceptable global standard for EHRs would improve generalizability and portability. This would involve greater promotion, and development, of standards for research such as TRIPOD [42] and, for data interchange, Health Level Seven International [75], and their further development to support ML/EHR applications. Future work could pay more attention to generalizability and interpretability, both of which need to be addressed prior to trialling implementation in the clinic. It is also worth investigating areas where performance can be improved, for example by including temporal sequence within the models, better selection of predictors and the use of non-anonymised/synthetic data. Our review suggests depression prediction using ML/EHRs is a worthwhile area for future development.

### Supplementary Information

**Additional file 1: Table S-1.** Studies excluded at full text stage with reasons.

## Data Availability

All data generated or analysed during this study are included in this published article [and its supplementary information files].

## Abbreviations

AUC-ROC: Area Under Curve – Receiver Operating Characteristic
ANN: Artificial Neural Network
ARM: Association Rule Mining
BRTLM: Bidirectional Representation Learning model with a Transformer architecture on Multimodal EHR
DNN: Deep Neural Network
EHR: Electronic Health Records
KNN: K Nearest Neighbours
LASSO: Least Absolute Shrinkage Selection Operator
LR: Logistic Regression
MLP: Multilayer Perceptron
M SEQ: Multiple-input multiple-output Sequence
NB: Naïve Bayes
NLP: Natural Language Processing
SVM: Support Vector Machine
XGBoost: eXtreme Gradient Boosting
