# Supplementary material for: Prediction and diagnosis of depression using machine learning with electronic health records data: a systematic review
Source: BMC Med Inform Decis Mak. 2023 Nov 27;23:271. doi: 10.1186/s12911-023-02341-x (PMC10680172; doi:10.1186/s12911-023-02341-x)
Supplement: Supplementary file 1 — Additional file 1: Table S-1. Studies excluded at full text stage with reasons. [file 12911_2023_2341_MOESM1_ESM.docx]

Table S-1 Studies excluded at full text stage with reasons

|  | **Study** | **Reason for exclusion** |
| --- | --- | --- |
| 1 | Camdeviren, H. A., Yazici, A. C., Akkus, Z., Bugdayci, R., & Sungur, M. A. (2007). Comparison of logistic regression model and classification tree: An application to postpartum depression data. Expert Systems with Applications, 32(4), 987–994. <https://doi.org/10.1016/j.eswa.2006.02.022> | Primary focus based on comparison of methods rather than disorder prediction using specific data relating to education/demographics of parents rather than EHRs. |
| 2 | Cho, C.-H., Lee, T., Kim, M.-G., In, H. P., Kim, L., & Lee, H.-J. (2019). Mood Prediction of Patients With Mood Disorders by Machine Learning Using Passive Digital Phenotypes Based on the Circadian Rhythm: Prospective Observational Cohort Study. Journal of Medical Internet Research, 21(4), e11029. <https://doi.org/10.2196/11029> | Circadian rhythm analysis, data collection via smartphone rather than EHRs sourced data. |
| 3 | Gokten, E. S., & Uyulan, C. (2021). Prediction of the development of depression and post-traumatic stress disorder in sexually abused children using a random forest classifier. Journal of Affective Disorders, 279, 256–265. <https://doi.org/10.1016/j.jad.2020.10.006>. | The sample used was specifically selected rather than being general population so not useful for broader prediction of depression. |
| 4 | Gong, J., Simon, G. E., & Liu, S. (2019). Machine learning discovery of longitudinal patterns of depression and suicidal ideation. PLOS ONE, 14(9), e0222665. <https://doi.org/10.1371/journal.pone.0222665> | Discriminating between depression and suicide ideation rather than predicting depression. |
| 5 | Hanauer, D. A., Ramakrishnan, N., & Seyfried, L. S. (2013). Describing the Relationship between Cat Bites and Human Depression Using Data from an Electronic Health Record. PLOS ONE, 8(8), e70585. <https://doi.org/10.1371/journal.pone.0070585> | Used EHRs to identify patients with cat bites who also had depression. Effectively identified bites as depression predictor not creation of a model to predict depression. |
| 6 | Jiménez-Serrano, S., Tortajada, S., & García-Gómez, J. M. (2015). A Mobile Health Application to Predict Postpartum Depression Based on Machine Learning. Telemedicine Journal and E-Health: The Official Journal of the American Telemedicine Association, 21(7), 567–574. <https://doi.org/10.1089/tmj.2014.0113> | Used specifically created data rather than general EHRs also specific to the development of a mobile phone application/interface. |
| 7 | Liberman, J. N., Davis, T., Pesa, J., Chow, W., Verbanac, J., Heverly-Fitt, S., & Ruetsch, C. (2020). Predicting Incident Treatment-Resistant Depression: A Model Designed for Health Systems of Care. Journal of Managed Care & Specialty Pharmacy, 26(8), 987–995. <https://doi.org/10.18553/jmcp.2020.26.8.987> | Predicting treatment resistance rather than depression. |
| 8 | Lyalina, S., Percha, B., LePendu, P., Iyer, S. V., Altman, R. B., & Shah, N. H. (2013). Identifying phenotypic signatures of neuropsychiatric disorders from electronic medical records. Journal of the American Medical Informatics Association, 20(e2), e297–e305. <https://doi.org/10.1136/amiajnl-2013-001933> | Focussed on autism, bipolar disorder, and schizophrenia rather than depression. |
| 9 | Menke, A. (2018). Precision pharmacotherapy: Psychiatry’s future direction in preventing, diagnosing, and treating mental disorders. Pharmacogenomics and Personalized Medicine, 11, 211–222. <https://doi.org/10.2147/PGPM.S146110> | Broad scope, not focused on predicting/diagnosing depression using EHRs but does consider applications of ML. |
| 10 | Mumtaz, W., Malik, A. S., Ali, S. S. A., Yasin, M. A. M., & Amin, H. (2015). Detrended fluctuation analysis for major depressive disorder. 2015 37th Annual International Conference of the IEEE Engineering in Medicine and Biology Society (EMBC), 4162–4165. <https://doi.org/10.1109/EMBC.2015.7319311> | Based on EEG data not deriving diagnosis from EHRs. |
| 11 | Perlis, R. H., Iosifescu, D. V., Castro, V. M., Murphy, S. N., Gainer, V. S., Minnier, J., Cai, T., Goryachev, S., Zeng, Q., Gallagher, P. J., Fava, M., Weilburg, J. B., Churchill, S. E., Kohane, I. S., & Smoller, J. W. (2012). Using electronic medical records to enable large-scale studies in psychiatry: Treatment resistant depression as a model. Psychological Medicine, 42(1), 41–50. <https://doi.org/10.1017/S0033291711000997> | Scope too broad. Model specific to treatment resistant depression identification, not prediction of depression. |
| 12 | Shim, M., Jin, M. J., Im, C.-H., & Lee, S.-H. (2019). Machine-learning-based classification between post-traumatic stress disorder and major depressive disorder using P300 features. NeuroImage: Clinical, 24, 102001. <https://doi.org/10.1016/j.nicl.2019.102001> | Discriminates between disorders rather than predicting depression also uses EEG data. |
| 13 | Taquet, M., Luciano, S., Geddes, J. R., & Harrison, P. J. (2021). Bidirectional associations between COVID-19 and psychiatric disorder: Retrospective cohort studies of 62 354 COVID-19 cases in the USA. The Lancet Psychiatry, 8(2), 130–140. <https://doi.org/10.1016/S2215-0366(20)30462-4> | Breadth of outcome disorders too large for inclusion, not sufficiently focussed depression. |
| 14 | Uyulan, C., Ergüzel, T. T., Unubol, H., Cebi, M., Sayar, G. H., Nezhad Asad, M., & Tarhan, N. (2021). Major Depressive Disorder Classification Based on Different Convolutional Neural Network Models: Deep Learning Approach. Clinical EEG and Neuroscience, 52(1), 38–51. <https://doi.org/10.1177/1550059420916634> | Discriminates between disorders rather than predicting depression, also uses EEG data. |
| 15 | Xiong, H., Zhang, J., Huang, Y., Leach, K., & Barnes, L. E. (2017). Daehr: A Discriminant Analysis Framework for Electronic Health Record Data and an Application to Early Detection of Mental Health Disorders. ACM Transactions on Intelligent Systems and Technology, 8(3), 47:1-47:21. <https://doi.org/10.1145/3007195> | Primarily concerned with the development of a specific technique for detection in general not a depression focussed study. |
| 16 | Yang, S., Bian, J., Sun, Z., Wang, L., Zhu, H., Xiong, H., & Li, Y. (2018). Early Detection of Disease Using Electronic Health Records and Fisher’s Wishart Discriminant Analysis. Procedia Computer Science, 140, 393–402. <https://doi.org/10.1016/j.procs.2018.10.299> | Breadth of outcome disorders too large for inclusion, not focussed on depression. |
| 17 | Yang, S., Zhou, P., Duan, K., Hossain, M. S., & Alhamid, M. F. (2018). emHealth: Towards Emotion Health Through Depression Prediction and Intelligent Health Recommender System. Mobile Networks and Applications, 23(2), 216–226. <https://doi.org/10.1007/s11036-017-0929-3> | Based on data collected via a mobile phone-based application. |
| 18 | Zimmerman, M., Balling, C., Chelminski, I., & Dalrymple, K. (2019). Symptom presence versus symptom intensity in understanding the severity of depression: Implications for documentation in electronic medical records. Journal of Affective Disorders, 256, 344–347. <https://doi.org/10.1016/j.jad.2019.05.073> | Relates to structure/content of EHRs, not depression prediction models. |
